# An intervention to support stroke survivors and their carers in the longer term (LoTS2Care): study protocol for the process evaluation of a cluster randomised controlled feasibility trial

**DOI:** 10.1186/s13063-018-2683-7

**Published:** 2018-07-11

**Authors:** Natasha K. Hardicre, Thomas F Crocker, Alan Wright, Louisa-Jane Burton, Seline Ozer, Ross Atkinson, Allan House, Jenny Hewison, Christopher McKevitt, Anne Forster, Amanda J. Farrin, on behalf of the LoTS2Care Programme Management Group

**Affiliations:** 1 Academic Unit of Elderly Care and Rehabilitation, Bradford Teaching Hospitals NHS Foundation Trust, Bradford, UK; 2 Leeds Institute of Health Sciences, University of Leeds, Leeds, UK; 3 Centre for Health Services Research, Leeds Institute of Health Sciences, University of Leeds, Leeds, UK; 4 School of Population Health & Environmental Sciences, King’s College London, London, UK; 5 Academic Unit of Elderly Care and Rehabilitation, Leeds Institute of Health Sciences, University of Leeds, Leeds, UK; 6 Clinical Trials Research Unit, Leeds Institute of Clinical Trials Research, University of Leeds, Leeds, UK

**Keywords:** Process evaluation, Complex intervention, Self-management, Stroke, New Start, Logic model, Outcomes chain, Interviews, Observations

## Abstract

**Background:**

Whilst pathways relating to the early stages of stroke care have become well established, strategies for longer-term care are less developed and longer-term outcomes remain poor for many stroke survivors. *New Start*, a complex intervention that includes needs identification, exploration of social networks and components of problem-solving and self-management, was designed to improve stroke survivors’ quality of life by addressing unmet needs and increasing participation. It is delivered approximately 6 months post-stroke by trained staff (facilitators). We are currently undertaking a cluster randomised feasibility trial of the New Start intervention with an embedded process evaluation, which is an important component of the design and testing of complex interventions as it provides an understanding of how interventions are delivered and function in different settings.

**Methods/design:**

This mixed methods process evaluation will explore the degree to which New Start is implemented as intended, the impact of context on intervention delivery and the acceptability of the intervention for stroke survivors, their families and practitioners. It will include non-participant observation of facilitator training and intervention delivery. Interviews with stroke survivors, facilitators and other relevant staff (including administrators and managerial staff) will be undertaken. Qualitative data from interview transcripts, facilitator reflections and observational field notes will be analysed thematically alongside numerical data documenting intervention delivery collected as part of the trial.

**Discussion:**

This process evaluation will identify factors that aid and impede implementation of the New Start intervention and improve understanding of this novel approach to longer-term stroke care.

**Trial registration:**

ISRCTN Registry, ISRCTN38920246. Registered on 22 June 2016.

## Background

The poor longer-term outcome for patients and their carers after stroke has been well documented [1, 2]. We are conducting a programme of research to develop an evidence-based and replicable longer-term care strategy for stroke survivors and their carers (the LoTS2Care programme). Through a structured process, we have developed an intervention, called *New Start*, that is designed to improve stroke survivors’ quality of life by addressing unmet needs and increasing participation. The LoTS2Care feasibility trial is a cluster randomised controlled trial (cRCT) of the New Start intervention, designed to gather data to inform the feasibility and acceptability of conducting a future definitive cRCT.

### New Start intervention

Our intent was to address the national guideline recommendation for a 6-month review of health and social care needs [3] with an evidence-based intervention. The development of New Start will be reported separately, but in summary, the intervention involves a trained facilitator eliciting a stroke survivor’s needs and mapping their social networks at approximately 6 months post-stroke. An open process seeks to enable stroke survivors to address their identified needs and includes components of problem-solving and self-management. The implementation of the intervention is supported by a training package that trains individuals within a service unit to become New Start facilitators.

#### Key intervention components

##### New Start training course

Stroke services randomised to the intervention arm will identify facilitators to be trained in the intervention. It is anticipated that the New Start facilitators will have experience in one of the following roles: nurse, physiotherapist, occupational therapist, health and well-being practitioner, or will have other allied health professional training, and will have stroke-specific knowledge or training.

The facilitators will be trained in delivering New Start in the weeks preceding trial commencement. The initial training will consist of a 2-day training course during which facilitators will learn relevant theory about a self-management approach and communication skills. They will also learn about the intervention and how to deliver it to stroke survivors, with the opportunity to observe aspects of the intervention being delivered and to practise these skills. The facilitators will then practise delivering the intervention in their services. A further training day will take place approximately 3 to 6 weeks after the initial course and will provide facilitators with the opportunity to discuss progress and challenges with colleagues in a supportive environment. Purposively designed training records will be kept to document the training received by each facilitator.

The New Start facilitators will be assessed for competency in the delivery of the New Start intervention approximately 16 weeks after completing the initial training course, through a review of purposively designed documents to record intervention activity (termed activity records), reflective reports, interviews and observation. This will coincide with trial recruitment commencing.

##### New Start supported self-management sessions with stroke survivors

The New Start intervention will be offered to all stroke survivors within the stroke services allocated to the intervention arm and who are approximately 6 months post-stroke. The New Start intervention consists of an initial face-to-face meeting, at the stroke survivor’s home or in clinic, with a trained facilitator. It seeks to help stroke survivors identify any unmet needs they may have and then to work with them to address these needs. A leaflet providing a list of common problems faced by stroke survivors (termed a priming tool) will be sent out with an appointment letter in advance to highlight potential topics for discussion (unmet needs) and it invites the survivor to add their own. At the meeting, these issues and needs will be discussed (whether noted on the priming tool or not) and a supported self-management approach introduced. The process involves facilitated action-planning, goal-setting, and review, all of which are supported by New Start materials, a set of worksheets developed during previous phases of the LoTS2Care programme. At each stage, participants are encouraged to see those in their social network as resources to help in this process. Booklets containing information about stroke and useful contacts are also available and can be provided to stroke survivors as appropriate. Stroke survivors can have as many meetings with the facilitators as required. It is anticipated that most stroke survivors will have at least three visits and support may also be provided via other means (e.g. by phone or email).

### Feasibility trial

The protocol for the trial has been described in detail in a separate paper [4]. Briefly, the trial involves randomisation of ten UK-based National Health Service (NHS) stroke services on a 1:1 basis, either to implement the New Start intervention in their service, with delivery to the whole stroke population (intervention arm), or to continue with usual care (control arm). Approximately 200 stroke survivors across all the stroke services will be recruited into the trial. They will complete outcome assessments at baseline, and three, 6 and 9 months post-recruitment. In keeping with the cluster design, all stroke survivors within the intervention arm services will be offered the opportunity to receive the New Start intervention when they are 6 months post-stroke, independent of their participation in the completion of outcome measures as part of the trial.

This paper describes the protocol for the process evaluation embedded within the LoTS2Care feasibility trial, which has been designed in accordance with the Medical Research Council guidance for conducting process evaluations of complex interventions [5, 6].

### Process evaluation development

The design for the process evaluation was developed through an iterative process including:

- Further articulating the theoretical models of the intervention and implementation
- Considering the relevant objectives for a process evaluation in the context of this feasibility and cRCT
- Considering possible data sources and cross-referencing with the objectives
- Developing a feasible protocol with consideration for the available resource and compatibility with the trial and implementation work.

A pragmatic approach was taken to ensure results were applicable to the needs of this feasibility trial and intervention development. We drew on principles of evaluation frameworks (including realist evaluation [7, 8], programme theory [9] and Grant’s framework [10]), a taxonomy of factors that influence implementation (the Consolidated Framework for Implementation Research [11]) and a theory of the work done in implementation (normalisation process theory, NPT [12]).

**Fig. 1:**
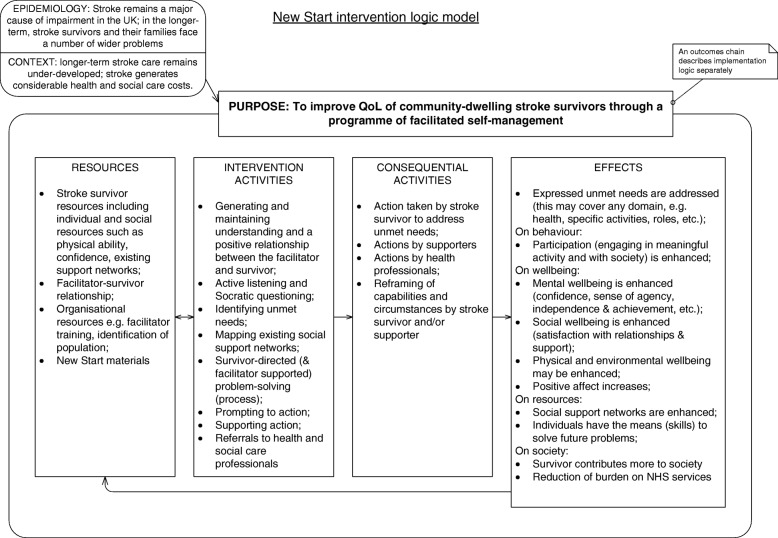
New Start intervention logic model. QoL quality of life

To articulate the theoretical model of the intervention and its implementation further, two authors (NH and TC) utilised the research findings from the prior intervention development work, alongside discussions with the research team exploring their understandings of the intervention and their intended strategy for implementation. Following this, the logic of the developed intervention was explicated in a diagram (Fig. 1) and accompanying explanatory text.

**Fig. 2:**
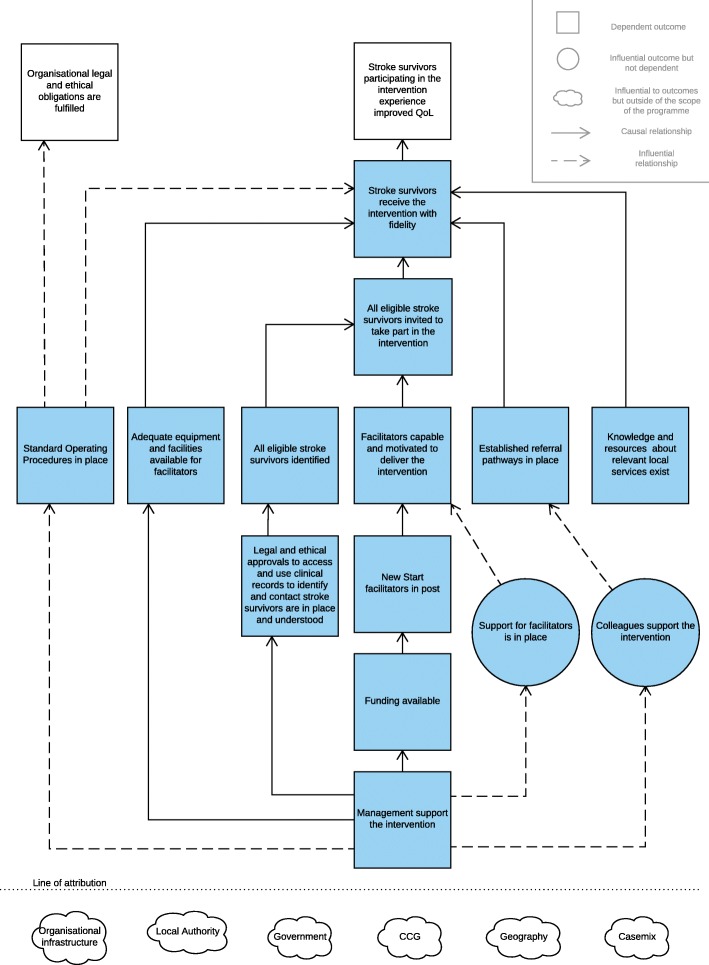
New Start outcomes chain. CCG Clinical Commissioning Group, QoL quality of life

In addition, an outcomes chain [9] was developed to represent diagrammatically a theory of change, i.e. what must be achieved in implementing the intervention to bring about the overall outcome — improved quality of life and increased participation for stroke survivors (Fig. 2). Each box contains an outcome (of implementation)^1^ that is related to other outcomes in the chain. Some of these relationships are stronger than others. Some outcomes are dependent^2^ on the outcome preceding it, for example, the successful appointment of New Start facilitators is dependent on funding being available. However, other outcomes are not dependent on previous outcomes but are importantly influenced by them, for example, facilitators being capable and motivated to deliver New Start may be more likely in the presence of management support. In all cases, achieving any given outcome does not mean that the following outcome will be achieved. Rather, it suggests that it can be (dependent relationship) or is more likely to be (influential relationship).

A matrix to accompany the outcomes chain (an example for one of the outcomes is provided in Table 1 for illustrative purposes) was also developed to describe details about success criteria for each outcome and factors that may aid or impede its achievement [9]. The process evaluation described in this protocol will be informed by these documents as a sensitising framework for the project and will examine the utility of the theory that underpins the design of the intervention and its implementation [10].

**Table 1:** Outcomes chain matrix (for one outcome as an illustrative example)

Outcome from outcomes chain	Success criteria	Programme factors	Non-programme factors	Activities (A) and principles (P)	Inputs and resources: financial (£) and human (☺)	Monitoring and assessment measurement
New Start facilitators are in post	Someone is employed by a stroke service to deliver the New Start intervention as a facilitator for 12 months. A facilitator is: • Willing to support stroke survivors through a programme of supported self-management (the New Start intervention) • Willing and able to attend the New Start training course • Employed in this role for a minimum of 2 days per week (0.4wte) A facilitator has some stroke-specific knowledge that includes the causes and short- and long-term consequences of a stroke.	Whether: Stroke services are given guidance on the desired attributes required of a New Start facilitator, and on the minimum time required for the delivery of the intervention.	Whether: Stroke services are willing or able to recruit a facilitator according to the guidance given to them. Facilitators can attend the training course.	A: LoTS2Care team provide guidance to stroke service and managers about the desired attributes of New Start facilitators. A: LoTS2Care team provide the dates of the New Start training course to stroke services prior to recruitment. A: Stroke services identify or recruit a facilitator following local processes.	£ Funding (site) – to employ a facilitator ☺ Management – to recruit an appropriate facilitator ☺ Human resources personnel – to support the employment process ☺ LoTS2Care team – to provide guidance about the facilitator role and dates for the training course	Interviews with managers to ask if enough guidance was given to enable them to take appropriate action, and to check whether a facilitator is in place according to the success criteria.

LoTS2Care is a feasibility randomised controlled trial. A key component of feasibility is the implementation of the intervention and therefore, much of this process evaluation will focus on exploring the ways in which New Start is implemented across the five intervention sites. In terms of Grant’s framework, these are the domains: delivery to clusters, response of clusters, recruitment and reach of individuals, and delivery to individuals [10]. Data collection and analysis will focus on exploring the outcomes contained in the blue boxes in Fig. 2, because these are the outcomes directly associated with implementation. This process evaluation will, however, collect and access limited data from control sites to make some comparisons between each of the sites in the trial. It will also look at the response of individuals: the ways in which the New Start intervention is received by the target population (stroke survivors) and what they do as a result. These latter activities will primarily inform the feasibility of data collection methods for a process evaluation of a future effectiveness trial.

### Aims and objectives

Our primary aim is to gain an understanding of how New Start is implemented and received by stroke survivors in a range of settings to inform the optimisation of its future design and evaluation. The objectives of this process evaluation are to:

1. ***Assess implementation fidelity.*** This will involve exploring whether the intervention was delivered to stroke survivors with fidelity, but the focus will be on assessing implementation processes and will be informed by strategies within the Borelli framework [13]. This framework provides guidelines and strategies for assessing and monitoring treatment fidelity across five domains: study design, provider training, treatment delivery, treatment receipt and treatment enactment.
2. ***Explore and clarify causal assumptions regarding implementation.*** This will involve looking at whether the activities and outcomes proposed in the logic model (e.g. funding available, New Start facilitator in post, and facilitators capable and motivated to deliver the care strategy) operate as intended, whether outcomes relate to one another as anticipated and which outcomes in the outcomes chain are necessary and which are redundant. It will also involve identifying factors that disrupt or facilitate activity or outcome achievement. For example, do unspecified or unanticipated activities contribute to outcome achievement, or do any activities result in unintended consequences?
3. ***Investigate the contextual factors associated with variations in intermediate outcomes between sites.*** This will involve a qualitative exploration of which contextual factors affect the implementation of the intervention in a site and which contextual factors are affected by the implementation of the intervention. It will also involve exploring the usual care provided in control sites.
4. ***Explore the views, perceptions and acceptability of the intervention to facilitators, stroke survivors and carers.*** This will involve exploring facilitators’, stroke survivors’ and carers’ views of the intervention and whether they find it useful and acceptable.
5. ***Test and refine methods of data collection and interrogation in preparation for a process evaluation alongside a future effectiveness trial.*** This will involve exploring whether we are able to generate and access the data required for this work, and whether the proposed methods are sufficient to allow fidelity and impact to be assessed in a process evaluation alongside a future effectiveness trial. We also intend to identify specific elements of data collection and interrogation that would optimise the future process evaluation.

These objectives have guided the data collection and data accessing processes. The process evaluation will, however, remain sufficiently flexible to allow for emergent questions to be addressed if necessary.

## Methods/design

### Data collection methods

Data collection will use a mixed methods approach, combining interviews, non-participant observation, documentary analysis, numerical data documenting intervention activity and data from public databases. The researchers undertaking the process evaluation did not design New Start nor will they be involved in implementation or trial procedures.

Table 2 summarises the different types of data that will be generated and used to address the process evaluation objectives.

**Table 2:** Summary of data to be generated and used to address the process evaluation objectives

Objectives	Data collection method	Data generated
1. Assess implementation fidelity	• Non-participant observation of intervention training • Self-report by facilitators • Non-participant observation of intervention delivery (including work shadowing) • Interviews with facilitators and relevant site staff • Documentation of intervention activity	Field notes Documents: structured reflective reports, SEPSS forms and NPT toolkit reports Documents: activity records. Transcripts.
2. Explore and clarify causal assumptions	• Non-participant observation of intervention training • Self-report by facilitators • Interviews with facilitators and relevant site staff	Field notes Documents: structured reflective reports, SEPSS forms and NPT toolkit reports Transcripts
3. Investigate the contextual factors associated with variations in intermediate outcomes between sites	• Site observation • Interviews with facilitators and relevant site staff • Structured site surveys (including interviews) • Documentation of intervention activity, facilitator activity and usual care • Data from public databases	Field notes Documents: site surveys Documents: activity records Database records. Transcripts.
4. Explore the acceptability of the intervention to facilitators, stroke survivors and carers	• Non-participant observation of intervention training • Self-report by facilitators • Non-participant observation of intervention delivery • Interviews with stroke survivors and carers • Interviews with facilitators	Field notes Documents: structured reflective reports, SEPSS forms and NPT toolkit reports Transcripts
5. Test and refine methods of data collection and interrogation in preparation for a process evaluation alongside a future effectiveness trial	• Non-participant observation of intervention training • Self-report by facilitators • Site observation • Non-participant observation of intervention delivery • Interviews with stroke survivors and carers • Interviews with facilitators and relevant site staff • Structured site surveys (including interviews) • Documentation of intervention activity, facilitator activity and usual care • Data from public databases • Researcher reflective diary (including difficulties log)	Field notes Documents: structured reflective reports, SEPSS forms and NPT toolkit reports Documents: site surveys Documents: activity records Database records Documents: diary entries and logs. Transcripts.

*NPT* normalisation process theory, *SEPSS* Self-Efficacy and Performance in Self-management Support instrument

#### Data collected by the process evaluation researchers

##### Non-participant observation of intervention training (addressing objectives 1, 2, 4 and 5)

This will include observing and recording the training delivered to facilitators and whether this was consistent with what was intended by the LoTS2Care implementation team. It will also include exploring the extent to which facilitators engage with the training process and how they seek to understand the intervention and their role in delivering it within their services. During the actual training sessions, the process evaluation researchers will not participate in any discussions or ask questions of the facilitators. During suitable breaks between sessions, however, the researchers may seek clarification or request further detail about something that has been observed to understand better what has been seen. Data generated through this observation will seek to explore the extent to which training was delivered as intended, together with how the training was received by facilitators. In addition to observation of training sessions, the process evaluation researchers will also look at the training records of each facilitator to gain a picture of what training has occurred within each site. They will record on standardised pro-formas what aspects of the training package each facilitator has completed and when.

##### Self-report by facilitators (addressing objectives 1, 2, 4 and 5)

Facilitators will be asked to complete the Self-Efficacy and Performance in Self-management Support instrument (SEPSS) [14] prior to their training, after both the initial training course and further training day, and again at the end of the trial. The completed forms will be analysed to gain an understanding of how facilitators view their capacity and current performance in delivery of self-management support to stroke survivors.

Facilitators will also be asked to complete the NPT toolkit [12, 15] during the early stages of implementation and then every 3 months until the end of the trial. This is an interactive online tool that can be used to help people think through problems in the implementation of an intervention. The toolkit is available from http://www.normalizationprocess.org/‌npt-toolkit/ and requires respondents to evaluate how the people and organisation involved in implementation are engaging in the work (sense-making, cognitive participation, collective action and reflexive monitoring). Upon completion, a report is generated, which will be shared with the LoTS2Care implementation team. The reports will be analysed to help in understanding the perspectives of key personnel about the implementation process.

As part of their ongoing training and development, facilitators will also be asked to participate in reflective practice. Facilitators will be asked to submit self-reflection reports monthly, which will be supported by a reflective framework created by the LoTS2Care implementation team. The framework will provide guidance on what types of questions the facilitators may consider, without becoming overly prescriptive. These reflective reports will be used to explore the implementation and delivery of the intervention and the responses of facilitators to this work. The reports may also provide helpful information about the responses of the organisation and of those receiving the intervention.

##### Site observation (addressing objectives 3 and 5)

There is a benefit in collecting naturally occurring data when seeking to understand an environment or context [16]. For this reason, prior to implementation of the intervention, the process evaluation researchers will undertake 1 day of non-participant observation within each intervention site. These observations will increase their understanding of what the organisational unit does, how it does it and why, to inform their understanding of usual care. This will enable the researchers to document change. During the observations, researchers will not seek to become involved in conversations, meetings or interactions, so as not to influence local processes, but may seek clarification about what has been observed through conversation at an appropriate later time. In addition to field notes, researchers will document what kinds of services are provided and to whom, how people are offered access to these services, what different roles exist within each of the sites and why, and how decisions are made within the organisation as a whole and on a day-to-day basis.

##### Non-participant observation of intervention delivery (addressing objectives 1, 4 and 5)

We will observe the intervention being delivered as it offers a direct view of what each facilitator does, together with how the intervention is received by stroke survivors and their supporters [17–19]. Observation may include informal conversation with the people observed to contextualise and explain what has been observed, though such informal conversation will not occur during one-to-one interactions between facilitators and stroke survivors/carers. A purposively designed sensitising framework (observation guide) will be used to guide field notes taken during these observations.

Opportunity sampling will be employed to observe intervention delivery. The intention is to observe at least one face-to-face encounter for each facilitator during both the establishing phase (between initial training and facilitators being assessed as competent) and delivery phase (between facilitators being assessed as competent and the end of the trial). Each stroke survivor will have their care observed only once. Because sampling is opportunistic, there are no inclusion or exclusion criteria for stroke survivors and carers taking part in observed sessions.

Alongside observation of the intervention delivery, a process evaluation researcher will accompany a facilitator during their day to gain insight into their working patterns, the ways in which they interact with other colleagues in the stroke service, and the administrative tasks and other duties that they do around intervention delivery. During the work shadowing, the process evaluator will capture data by taking field notes. The data collected during work shadowing will be used to tailor semi-structured interview guides for each of the facilitators.

##### Interviews with stroke survivors (addressing objectives 4 and 5)

Within the intervention sites, we will undertake semi-structured interviews with stroke survivors and their carers and supporters (where present and willing to participate). These interviews will explore their experiences of participating in the New Start intervention. Researchers may also, with agreement, retrospectively review and discuss intervention materials given to stroke survivors and carers to understand their use in practice. A topic guide will be devised drawing on our previous intervention development and piloting work. Interviews will aim to capture what stroke survivors and carers did during the meetings with facilitators (i.e. how many meetings there were, the duration of these meetings, and what actions occurred during the meetings); what stroke survivors and/or carers did as a result of participating in the intervention; whether, how and to what extent the intervention impacted the lives of stroke survivors and/or carers; alongside their experiences of participating in the intervention as a whole, including their experience of, and perspective about, the facilitators.

Stroke survivors will be purposively sampled to achieve a diversity of informants with respect to the extent of intervention activity and the time since first activity using data from the activity records. Inclusion criteria for participation in the interviews are: individuals must have had (or be caring for someone who has had) a stroke, they must be living in the community and they must have been approached to take part in the New Start intervention. Only stroke survivors who are not participating in the trial will be approached for interview to avoid the effects of interviews and intervention delivery being confounded. Approximately 25 stroke survivors and their carers or supporters—approximately five from each of the active sites—will be interviewed.

Following appropriate informed consent procedures, stroke survivors and their carers or supporters will be given the option of being interviewed together or separately. We expect that individuals will be interviewed only once. To include stroke survivors who have varying degrees of ability to communicate, steps will be taken to adapt the interview methods, for instance by using pictures, adapting the topic guide or writing down key words. Photographs of the facilitator(s) may also be shown to stroke survivors to aid their memory of the intervention where appropriate. These interviews will be conducted in a quiet, private area (usually the stroke survivor’s own home) and, with participants’ permission, be audio recorded. Demographic data including participants’ age group, gender, ethnicity, living arrangements and the relationship between the stroke survivor and carer (where participating) will be collected from each participant at the time of the interview for sample characterisation.

##### Interviews with New Start facilitators and relevant site staff (addressing objectives 1, 2, 3, 4 and 5)

At the end of the intervention implementation period, following appropriate informed consent procedures, we will conduct semi-structured interviews with all of the facilitators and with any additional site staff involved in implementation of the intervention (including administrators and managerial staff). Semi-structured topic guides will be utilised but will be sufficiently flexible to allow respondents to introduce any issues they consider relevant. The researchers will have gathered data from and about the services throughout the trial, so the topic guide will be devised close to when the interviews are undertaken, so that it can be tailored to each individual and reflect the context within which they work. Nonetheless, there will be core questions that each person will be asked, and these will be informed by previous work and the literature [20, 21]. Questions are likely to explore facilitators’ experiences of the training package and whether they felt adequately prepared to deliver the care strategy; their experiences of delivering the care strategy; the impact of the organisational context on how the strategy has been implemented and delivered (e.g. the development of the intervention in their service, links with other staff members, organisations and departments, and the flow of survivors through the service); and their insights into the impact the care strategy has had on their own practice, the organisation and for recipients. These individual interviews will be conducted in a quiet, private area—likely to be at the individual’s place of work—and, with participants’ permission, be audio recorded. Demographic data including participants’ age group, gender and professional background will be collected at the time of interview for sample characterisation.

#### Additional data

##### Structured site surveys (addressing objectives 3 and 5)

As part of the feasibility trial, details of current service provision will be collected by structured service surveys to enable usual care and the wider context of the service to be described. This survey will be repeated by post or through interviews and site visits at further time points throughout the trial to ensure any service and staffing changes are captured.

##### Documentation of intervention activity, facilitator activity and usual care (addressing objectives 1, 3 and 5)

As part of the feasibility trial, structured activity records will be used to capture specific information about New Start activities delivered to stroke survivors (including associated administrative and logistical work), other work that facilitators do (such as team meetings and training), and usual care provided by the service. Data includes time of occurrence, duration, specific activities conducted and who was involved. This information will be incorporated into assessments of intervention fidelity.

##### Data from public databases (addressing objectives 3 and 5)

Data from national sources of anonymous data, such as the Sentinel Stroke National Audit Programme, Clinical Commissioning Group Outcomes Indicator Set and census data provided by the UK Data Service, will be accessed and incorporated to describe and contextualise the services.

##### Researcher reflective diary (addressing objective 5)

Throughout the data collection period, the process evaluation researchers will keep a reflective diary to document their experiences of collecting data and will report any specific difficulties on a log. These will be analysed to explore whether the proposed methods of collecting data are sufficient to allow fidelity and impact to be assessed.

### Analysis

Recorded interviews will be transcribed verbatim and anonymised, and managed alongside anonymised observational field notes and records, facilitators’ reflective reports and site surveys using the qualitative data analysis tool QSR NVivo (version 10.0). Familiarisation with the data will be followed by data reduction, during which the researcher will engage in ‘selecting, focusing, simplifying, abstracting, and transforming the data’ [22] to identify patterns and themes within and between sets of data, thereby making sense of them and generating descriptions and explanations relevant to the phenomena being explored.

Standard approaches to demonstrating trustworthiness and quality in qualitative research will be used, including clearly documenting the research process (methods, analysis and any problems encountered and solutions found), transparently developing interview topic guides in light of ongoing analysis, documenting the contextual features in which the research was carried out, exploring deviant cases and alternative explanations, and discussing emerging findings among the process evaluation team. Moreover, the researchers will keep a reflexive diary [19, 22–24].

Data from the activity records will be entered into a database. These data will be analysed with descriptive statistics such as count, proportion, mean, median, standard deviation and interquartile range for each site and facilitator, and represented in graphs as appropriate.

### Ethical considerations

Written informed consent will be obtained from all study participants. For stroke survivors who lack capacity to give consent (e.g. due to cognitive difficulties associated with their stroke), consultee declaration will be sought from someone who is well placed to make a judgement on the stroke survivor’s wishes. The consultee will be advised to set aside their own views and provide advice on the stroke survivor’s participation in the research, taking into consideration the person’s wishes and interests. An information sheet advising them on their potential role and their right to refuse will be provided. Capacity to provide consent will be assessed by the researcher during the conversation with each potential participant before consultee declaration or consent is obtained. The research team has previous experience of working with and interviewing people who lack capacity and who have cognitive impairments. For those who are unable to read or sign the consent form due to impairments, but who have capacity to consent, the consent procedure will be witnessed (the witness may be the caregiver or significant other).

When seeking consent to observe intervention delivery, if there are several people present (e.g. the stroke survivor and a relative) and some decline consent while some provide consent, the researcher will seek to understand their reasons for declining and obtain agreement for one of the following options: no observation is conducted, the observation is conducted but nothing about those declining consent is recorded, or the observation is conducted and the people who declined consent are not present. The researcher will be led by the will and needs of the survivor and carer and will not seek to impose observation on anyone who is unwilling. We believe this will provide the appropriate balance between an individual’s right to participate in the research and another’s right to refuse participation.

The right of potential participants to refuse consent without giving reasons will be respected at every stage of the research process. All data will be kept confidential and stored securely.

#### Safeguarding of Adults

It is possible that, during discussions, stroke survivors may disclose information to the researcher, or the researcher may have concerns, that the individual may be experiencing abuse or is at risk of abuse. In such circumstances, the researcher will follow the relevant NHS Trust’s safeguarding adults policy (or equivalent document).

## Discussion

The process evaluation described here has been designed to explore the degree to which New Start*,* a newly developed component of a longer-term stroke care strategy, is implemented as intended, the impact of context on intervention delivery and the acceptability of the intervention for stroke survivors, their families and practitioners. Process evaluation is recommended and regarded as good practice in trials of complex interventions such as this, which involve several interacting components, including some element of behaviour change [10, 25]. Such evaluations enable us to open the black box of complex interventions, providing insight into how interventions are delivered on the ground and the underlying processes influencing them [10].

There is little guidance for process evaluations in the context of feasibility trials and relatively few published examples. We have incorporated several highly structured data collection elements and have also included less structured elements, such as field notes from observations and semi-structured interviews with a range of stakeholders, to help us identify the most appropriate factors to evaluate in a process evaluation alongside a future effectiveness trial.

Our process evaluation has been designed to focus on answering clear questions around implementation fidelity, contextual factors, feasibility of data collection, intervention acceptability and causal assumptions based on a pre-specified theory of change and logic model. The findings from this in-depth evaluation will be invaluable in the interpretation of outcomes from the feasibility cRCT and will inform the future optimisation of the New Start intervention and its definitive evaluation.

## Abbreviations

CCG: Clinical Commissioning Group
cRCT: Cluster randomised controlled trial
NHS: National Health Service
NPT: Normalisation process theory
REC: Research ethics committee
SEPSS: Self-Efficacy and Performance in Self-management Support instrument

## Footnotes

1 In this sense, an outcome is any positive state that should be achieved as part of the implementation and can include the results of work done or a structure being in place (an outcome of implementation), rather than specifically a clinical or health outcome. The term outcome is used in the wider evaluation field to emphasise the focus of a theory of change (what the results or outcomes of the change should be) rather than a theory of action (what should be done to bring about the change).

2 In a different context, one outcome may not be dependent on the outcomes presented here. However, in this context the dependent relationships proposed here are the expected ways in which outcomes will be achieved, given the other outcomes specified alongside them.
